# Exploring the Beliefs of Latine Mothers and Fathers from Diverse Backgrounds About the Role of Mobile Devices in Their Parenting Experiences

**DOI:** 10.3390/bs15081139

**Published:** 2025-08-21

**Authors:** Wendy Ochoa

**Affiliations:** College of Education, San Diego State University, San Diego, CA 92182, USA; wochoa@sdsu.edu

**Keywords:** parenting, mobile screen technologies, Latine, preschool

## Abstract

This study examined how a socioeconomically and linguistically diverse sample of Latine mothers (*n* = 20) and fathers (*n* = 20) of children under age five perceive the role of mobile screen technologies in their parenting experiences, using semi-structured interviews. The results show seven key themes that capture how parents believed these devices had both supported and hindered their parenting. These themes were organized into three overarching functions that reflect how mobile screen technologies were viewed and used by Latine mothers and fathers from socioeconomically and linguistically diverse backgrounds: (1) enabling access to parenting resources (i.e., access to information and social support), (2) shaping parent–child interactions (i.e., facilitating teaching, promoting bonding, and disrupting interactions), and (3) influencing emotional regulation and wellbeing (i.e., aiding or complicating behavior management and contributing to parental stress and relief). While these functions were largely consistent across participants, notable differences emerged by gender, language, and income—most prominently in relation to the parents’ levels of formal schooling.

## 1. Introduction

Since the release of the first iPhone in 2007, mobile screen technologies—such as smartphones and tablets—have become deeply embedded in the daily lives of most families in the United States (U.S.; Pew Research Center, 2024). Today, 96% of U.S. households own at least one smartphone and 75% also own at least one tablet that connects to the internet (Mann et al., 2025). The widespread adoption of these devices is likely due to their portability and multifunctionality—including instant access to the internet, contacts, social media, and entertainment (Ochoa & Reich, 2020). These features have enabled diverse families, including those with young children, to integrate mobile screen technologies into their daily routines in ways that align with their unique goals, needs, and contextual realities (Campbell et al., 2014; Katz, 2002). For instance, a father with a hybrid job might use his smartphone to attend work meetings virtually from home or the park to spend more time with his child, while another father with similar goals but more rigid work constraints may use his device to video call his child during work breaks. These examples highlight how mobile devices can be leveraged to navigate structural and logistical constraints in pursuit of parenting aims.

Indeed, a still limited but growing body of research suggests that mobile screen technologies have significantly influenced—and in some cases transformed—key aspects of parenting. These shifts have garnered widespread interest among various early childhood stakeholders, including researchers, educators, policymakers, and families, due to their potential implications for young children’s development and wellbeing. While many of these shifts reflect the agentic and creative ways in which parents leverage mobile screen technologies to support various dimensions of parenting, such narratives remain largely absent or underrepresented in both research and broader public discourse (e.g., Lauricella et al., 2018; Ochoa & Reich, 2020). Instead, dominant narratives have primarily emphasized concerns about families’ inappropriate use of mobile screen technologies and the associated risks to children’s development and wellbeing. These accounts often highlight patterns such as parental absorption with their devices—linked to fewer and lower-quality parent–child interactions—parents’ reliance on screens for managing their children’s behavior, and inadequate monitoring of children’s screen time and content, many of which have been associated with poor learning, language, and socioemotional child outcomes (Kildare & Middlemiss, 2017; McDaniel & Radesky, 2018). This focus has led to necessary and valuable efforts aimed at raising awareness and developing evidence-based media use recommendations for families with young children (Council on Communications and Media, 2016; reaffirmed 2022). However, within these narratives, ethnoracially minoritized parents with low incomes have been disproportionately portrayed as passive users of mobile screen technologies, lacking the knowledge—or motivation—to follow recommended screen media guidelines intended to maximize the benefits and minimize the risks of these technologies on their children’s learning and development (V. Rideout & Robb, 2020). These deficit-oriented portrayals reinforce long-standing, harmful stereotypes that depict Latine parents as inadequate or disengaged from their children’s learning (Valencia, 2010).

Although understanding and raising awareness about the risks associated with excessive and developmentally inappropriate screen use is critical, the dominant focus on Latine parents’ “misuse” of mobile technologies presents an incomplete and one-dimensional portrayal of screen use among Latine families. Moreover, current discourse often fails to situate families’ technology use within the broader structural conditions that contribute to patterns of excessive use (Kabali et al., 2015; Neuman & Celano, 2012). For example, extensive research shows that screen time is associated with income, with lower-income families averaging more time on screens than more affluent families (V. Rideout & Robb, 2020). Yet given that Black and Latine families are disproportionately affected by poverty due to systemic racism and structural inequalities, much of the existing research has inadvertently conflated race and ethnicity with “problematic” screen use (Kabali et al., 2015). As a result, the root of the issue has often been wrongly situated on families’ individual choices, rather than on the systemic conditions that constrain those choices—such as unequal access to affordable enrichment opportunities, safe and well-resourced neighborhoods, livable wages, and mental health support for families navigating the compounding negative effects of poverty and institutional racism (Neuman & Celano, 2012; Thompson et al., 2023).

Beyond misplacing the source of the problem on families rather than the existing U.S. systems, deficit narratives also obscure the potential positive ways in which low-income, ethnoracially minoritized parents may be leveraging mobile screens to support their parenting experiences within their contextual realities (Ochoa et al., 2024). Although research adopting non-deficit perspectives on families’ use of mobile screen technologies remains scarce, particularly those identifying as Latine, a handful of emerging studies have adopted more balanced frameworks and provided insights into the positive ways Latine families are also using mobile screen technologies. For instance, Ochoa and Reich (2020) conducted semi-structured interviews with socioeconomically and linguistically diverse Latine mothers and fathers of children under the age of five, revealing that parents actively employ strategies such as selecting appropriate content, setting time limits, and monitoring their child’s usage of these devices to ensure that they maximize their learning benefits while minimizing their risks. Similarly, Thompson et al. (2023) conducted semi-structured interviews with a sample of low-income, Latine parents of toddlers and found that they often used mobile screen devices to access parenting information, connect with their children, and facilitate learning. Despite these insights, there remains a critical need for further research focusing on socioeconomically and linguistically diverse Latine parents of young children. The majority of existing studies tend to portray Latine families as a homogeneous group, overlooking the significant variability in socioeconomic status, language practices, and other contextual factors that likely influence patterns of screen use. Such nuanced understanding is essential to comprehensively grasp the multifaceted ways in which families with young children engage with mobile screen technologies and to develop culturally and contextually informed guidelines around screen use (V. Rideout & Robb, 2020).

This study seeks to provide a more comprehensive understanding of mobile screen use within the highly diverse Latine community by centering the perspectives of socioeconomically and linguistically diverse Latine parents raising young children in Southern California. Using semi-structured interviews, this qualitative study explores how Latine mothers and fathers of young children perceive the role of mobile screen technologies and accompanying social media in their parenting—including how they believe these devices have both supported and hindered their parenting experiences. Latine parents of young children are the focus of this study, as early childhood represents a critical developmental period during which parenting practices are closely tied to children’s lifelong wellbeing (Center on the Developing Child at Harvard University, 2016).

### 1.1. Theoretical Framework

This study draws from Bronfenbrenner’s revised bioecological systems model (Vélez-Agosto et al., 2017), which underscores the central role of both parents and culture in shaping children’s development during early childhood. This developmental period is considered critical, as it is when proximal processes—the frequent and reciprocal interactions between a child and the adults and tools within their immediate environments, or *microsystems*—have the most significant influence on their development. At this stage, parents are typically the primary agents shaping children’s everyday environments, both directly through parent–child interactions and indirectly by structuring daily routines and largely determining the tools and activities available to their children across many microsystems such as the home and neighborhood spaces (e.g., parks and playgrounds; Bronfenbrenner, 2005). Importantly, Bronfenbrenner’s revised model emphasizes that parents’ childrearing beliefs, values, and practices are culturally and contextually situated (Fuller et al., 2015; Vélez-Agosto et al., 2017). In the context of this study, the ways in which parents interact with their children, organize routines, and structure their environments—including the tools they expose their children to, make available, or restrict—reflect their cultural values, available resources, and everyday realities. For example, a mother aiming to foster her child’s heritage language may intentionally seek out and co-view Spanish-language programs with her child on a smartphone or tablet.

Notably, smartphones and tablets have become some of the most salient cultural tools within many of children’s microsystems, including those they share with their parents (e.g., home, playgrounds; Radesky & Christakis, 2016). These devices frequently mediate interactions between children and people in these settings (e.g., parents, siblings), as well as between children and other objects in the environment (e.g., toys, books; Thompson et al., 2023). For instance, parents and children might jointly or independently use a smartphone at the park, or a parent may instruct a babysitter to allow tablet use only after dinner. These examples illustrate the ways in which parents play a central role in determining whether, when, and how mobile screen devices mediate their young children’s daily interactions and experiences (Radesky & Christakis, 2016). Given the widespread use of mobile devices among families with young children, it is critical to explore parents’ perspectives and practices related to these technologies. Such insights are essential for informing the development of digital media and screen guidelines that more accurately reflect the goals, beliefs, and lived realities of diverse Latine families.

### 1.2. The Current Landscape: Research on Mobile Screen Technologies and Parenting

To date, research examining how parents use and perceive mobile screen technologies and social media in relation to their parenting experiences remains limited—particularly among Latine parents of young children. However, emerging findings from studies with low-income Latine parents suggests that their experiences often reflect those previously documented among middle-class White families and families with older children. In both cases, mobile screen technologies appear to be described as a double-edged tool that can both support and hinder parenting (e.g., Ochoa & Reich, 2020; Radesky et al., 2016). Further, although the term “*parents*” is frequently used in the literature, existing studies have been disproportionately shaped by the perspectives of mothers—most often White and from middle- or upper-class backgrounds. Far fewer studies have included fathers, particularly ethnoracially minoritized fathers such as Latines. Unfortunately, but unsurprisingly, discussion of within-group variability in Latine mothers’ and fathers’ use and perspectives on mobile screen technologies remains virtually nonexistent.

These limitations are important to address for several reasons. First, Latines are the largest ethnoracially minoritized group in the U.S., yet they remain underserved by multiple institutions, including schools—an issue with serious implications for the future wellbeing and workforce of the nation (U.S. Census Bureau, 2023). Second, 67% of Latine children live in two-parent households (U.S. Census Bureau, 2023), and research in other domains of parenting highlights that Latine fathers make unique and meaningful contributions to their children’s development (Cabrera & Bradley, 2012). Third, the vast majority of Latine families have access to mobile screen technologies (Pew Research Center, 2017a; The Aspen Institute, 2023), and national reports indicate that Latines are the racial/ethnic group most reliant on smartphones to access the internet (Pew Research Center, 2017b). Researchers have attributed this trend to disparities in computer and broadband access, with 80% of White individuals having broadband access via a computer compared to 65% of Latines. In contrast, 25% of Latines rely solely on their smartphones for internet access compared to just 12% of White individuals (The Aspen Institute, 2023). Fourth, national data suggest that socioeconomic status and race/ethnicity shape patterns of screen use (Common Sense Media, 2017). Yet much of the research on ethnoracially minoritized families—particularly Latine families—has focused almost exclusively on low-income mothers (Common Sense Media, 2017; V. J. Rideout, 2014; Wartella et al., 2013). As a result, little is known about how key demographic factors—such as socioeconomic status and primary language(s) spoken—shape Latine mothers’ and fathers’ perspectives and practices around mobile device use.

The next sections review prior research exploring how mobile screen technologies have both positively and negatively influenced parenting among U.S. families with young children.

### 1.3. Mobile Screen Technologies: A Double-Edged Parenting Tool for Parents of Young Children

Mobile Screen Devices Disrupt and Enhance Parent–Child Interactions. Parent–child interactions are among the most widely studied aspects of parenting in the context of mobile screen technologies, particularly in families with young children. According to this body of research, mobile screen technologies seem to both hinder and enhance the quality of interactions between parents and their young children (Ochoa & Reich, 2020; Radesky et al., 2016). On the one hand, observational studies conducted anonymously in public settings, such as parks and restaurants, have often found that caregivers tend to engage in fewer verbal exchanges, initiate fewer interactions, and be less responsive to their young children when they use a mobile screen device compared to when they do not or compared to caregivers who refrain from using their devices during the entire observational period (Ochoa et al., 2020; Radesky et al., 2014). Similar patterns have been observed in controlled lab settings. For example, a study conducted among low-income mothers (72% White, 28% Latine) of six-year-old children found that those who spontaneously used a mobile device during a structured task with their child tended to talk less to their child than mothers who refrained from using their device entirely (Radesky et al., 2016). These findings have been supported by survey and interview studies disproportionately conducted among middle-class White mothers and, to a lesser extent, low-income, ethnoracially minoritized mothers. Specifically, most have reported struggling to set boundaries between work and family time, often feeling pressured to respond to work-related notifications immediately even while spending time with their children (Hiniker et al., 2015; Radesky et al., 2016).

On the other hand, a much smaller body of research suggests that mobile screen technologies are not solely a source of distraction to parent–child interactions. Evidence from experimental studies suggests that when used intentionally and appropriately, these devices can enhance the quality of parent–child interactions and contribute to positive child learning outcomes. For instance, lab-based studies involving predominantly White, middle-class mothers and children have found that co-viewing video content or co-using e-books—when both are educational and developmentally appropriate—as well as engaging in video chats (e.g., FaceTime), can foster high-quality parent–child interactions such as turn-taking and joint attention, both of which are associated with positive child outcomes during early childhood (Glick et al., 2022). These findings align with those found in two qualitative studies conducted among socioeconomically diverse Latine mothers and fathers of young children. For example, Thompson et al. (2023) interviewed 32 Spanish and English-speaking Mexican American mothers (*n* = 22) and fathers (*n* = 10) of toddlers from low-income households and found that many of them reported intentionally co-viewing content on TV and their mobile screen devices with their children to bond and engage with them. Similarly, Ochoa and Reich (2020) found that socioeconomically diverse Latine mothers and fathers of children under the age of five described intentionally co-using smartphones and tablets with their children to support the child’s learning by discussing the content on the screen and bridging knowledge gaps.

Altogether, emerging findings suggest that rather than simply hindering parent–child interactions, mobile screen devices can also serve as tools that extend and enhance the quality of parent–child interactions when used intentionally and appropriately.

### 1.4. Mobile Screen Devices Aid and Complicate Managing Children’s Behavior

Research evidence also suggests that mobile screen technologies can serve as a double-edged tool in managing children’s behavior for many parents. On one hand, findings from survey and interview studies among mostly middle-class, White and low-income, ethnoracially minoritized mothers indicate that parents report using mobile screen devices to occupy or calm their children down, particularly when other strategies, such as providing the child with a toy, do not work or are not viable (Radesky et al., 2016). Some families also describe relying on mobile screen devices to help their children relax before bedtime, stay occupied while running errands, or remain engaged while they complete household chores that could pose dangers for their young children, such as cooking (Radesky et al., 2016; Sergi et al., 2017). These findings have been corroborated by the handful of studies conducted among Latine mothers and fathers of young children (Thompson et al., 2023). On the other hand, many of these same parents also express concerns about their children becoming overly reliant on mobile screens for self-soothing and entertainment. Some worry that excessive screen use may hinder their children’s ability to develop independent coping skills, increasing their dependence on digital distractions (Ochoa & Reich, 2020; Thompson et al., 2023). These concerns are supported by research showing that children with socioemotional difficulties are more likely to be given a phone by their parents to help them calm down compared to children without such difficulties (Radesky et al., 2016). In sum, while many parents view mobile screen technologies as helpful tools for managing their children’s behavior—particularly when they are busy—they also recognize the risks of overreliance, highlighting the importance of intentional and balanced use.

### 1.5. Mobile Screen Devices Increase Access to Information and Misinformation

An emerging body of research suggests that mobile screen technologies have expanded parents’ access to both parenting-related information and social support networks. In a nationally representative survey of 494 socioeconomically diverse U.S. parents (47% fathers), mothers were more likely than fathers to report using platforms like Facebook to access parenting advice and emotional support (Duggan & Lenhart, 2015). However, only 30% of surveyed parents had a child under the age of five, and the majority identified as White (56%), with just 21% identifying as Latine—making it difficult to draw conclusions about the experiences of socioeconomically and linguistically diverse Latine parents of young children.

Although a growing body of research has explored how ethnoracially minoritized low-income mothers of young children utilize mobile screen technologies to access information, these studies have primarily focused on Black mothers. Far fewer studies have included Latine parents of young children, and the few that exist have largely sampled low-income, Spanish-speaking Latine mothers. For example, Criss et al. (2015) conducted focus groups in Spanish with 49 Latine mothers of children from conception to age two. Most of these mothers had less than a high-school education. Findings showed that participants generally trusting their healthcare providers for parenting and child-related information, but they also turned to Spanish-language websites like BabyCenter.com and selected social media platforms when they sought immediate parenting advice. These mothers reported cross-referencing multiple sources to validate the information they found but also expressed difficulty navigating misinformation and contradictory content online.

Similarly, in a two-part study with a sample largely composed of foreign-born, low-income, Spanish- and English-speaking Latine parents (82% mothers) of children between the ages of zero and nine, findings showed that parents used mobile screen devices to supplement—but not replace—offline family networks. Despite the accessibility of digital resources, many expressed confusion when confronted with conflicting advice and voiced skepticism about the reliability of online content, particularly in the absence of recommendations from trusted relatives or healthcare providers. For most of these parents, their offline family networks remained their primary source of parenting support. These parents also reported frequently using video calls, social media, and messaging apps to exchange parenting strategies and maintain connections with extended family. As a whole, the limited number of existing studies have overwhelmingly focused on mothers with low incomes and seem to indicate that while they value the instant access to parenting information their devices provide, they remain uncertain about the trustworthiness of information, particularly when they encounter conflicting advice.

### 1.6. Mobile Screen Devices Are a Source of Parental Relief and Stress

Although research is still limited, emerging mobile screen technologies have also been shown to exert both positive and negative effects on parental mental health. On one hand, many parents view mobile devices as helpful tools for multitasking and managing stress, especially during high-pressure periods such as the COVID-19 pandemic. Research by Reich et al. (2023) found that some parents used screens to occupy their children so they could decompress or complete necessary tasks, a pattern particularly common among parents facing financial strain or mental health challenges. On the other hand, screen use has also been linked to increased stress, guilt, and emotional exhaustion. Parents working remotely or in tech-heavy jobs often struggle to separate work and family time, which can lead to burnout (Hiniker et al., 2015; Kushlev & Dunn, 2019). Some report feeling judged for using devices around their children—whether by family members or other parents in public spaces (Mallawaarachchi et al., 2022). Moreover, the constant connectivity enabled by mobile technologies can lead to information overload and the pressure to be perpetually available, compounding parental stress. These findings underscore the double-edged nature of mobile screen technologies—not only in how they affect children, but also in how they impact the mental wellbeing of parents navigating the daily demands of caregiving.

### 1.7. The Present Study

While dominant discourse tends to pathologize the screen use of low-income and ethnoracially minoritized families, emerging research suggests that many Latine parents of young children engage with mobile screen technologies in intentional and adaptive ways—navigating their use within structurally constrained environments. Altogether, existing studies highlight that mobile screen technologies present both benefits and drawbacks for parenting. Parents use screens to supplement caregiving—whether by providing educational content, accessing parenting information, or staying connected with family—while also contending with concerns about misinformation, screen dependence, and disrupted parent–child interactions.

Yet most research to date has centered on White, middle-class mothers or low-income, foreign-born Latine mothers, often overlooking variation within the Latine community and excluding the voices of fathers. Moreover, many studies focus on parents of older children or use broad age categories that obscure the specific experiences of parents raising young children. To address these gaps, this qualitative study draws on semi-structured interviews with 40 socioeconomically (i.e., low-income and middle-to-high-income) and linguistically diverse Latine mothers and fathers of young children (ages 0–4 years). It explores how parents perceive mobile screen devices—and accompanying social media—as both supporting and hindering their parenting experiences.

## 2. Method

### 2.1. Participants

A total of 40 self-identified Latine parents of children under the age of five years old living in Southern California participated in this study throughout the years of 2018 and 2019. All mothers and fathers were intentionally recruited to be distributed equally across the low-income (*n* = 20) and middle-to-high income (*n* = 20) categories. Participants were recruited using three strategies: (a) referrals from individuals who were ineligible for a prior study but had expressed interest in participating in future research, (b) flyer distribution at community sites (e.g., local businesses, churches, grocery stores), and (c) snowball sampling. Eight participants (four couples) were from the same households but participated in interviews independently. Further, two couples had a low income and two couples had a middle-to-high income. The remaining 32 parents represented different families (i.e., were not couples). The next sections provide more detail on the demographic characteristics of the parents from each of the four groups, and a summary of these can be found in Table 1.

***Low-income mothers.*** Low-income mothers (*n* = 10) ranged in age from 22 to 36 years (M = 28.77, SD = 5.33). On average, they had two children (M = 2.1, SD = 1.37) and at least one child was under the age of five years old (M = 1.9 years, SD = 1.5). Descriptive statistics of the target children (i.e., those under the age of five years old) reveal that 21% were female and 79% were male. Sixty percent of the mothers had a high-school education or less, 20% had completed some form of college, and 20% had earned a Bachelor’s degree. Additionally, 60% of mothers in this income group were born in Mexico (*n* = 5) or Ecuador (*n* = 1), resulting in six of the ten mothers with low incomes being born outside of the U.S. These mothers had been living in the U.S. for an average of 15 years (M = 15.08, SD = 9.19). Nine of the ten mothers were married or living with someone and one mother reported being single. Finally, the majority of low-income mothers (70%) reported speaking both English and Spanish and 30% reported only speaking Spanish. Noticeably, two monolingual Spanish-speaking mothers had an elementary school education and one had a Bachelor’s degree; however, information about where this last mother completed her degree was not collected

***Low-income fathers.*** Low-income fathers (*n* = 10) ranged in age from 26 to 45 years (M = 31.50, SD = 6.28). On average, and similar to low-income mothers, this group of fathers had two children (M = 1.9, SD = 0.87) and at least one child was under the age of five years old (M = 2.0 years, SD = 1.2). From the target children (i.e., under the age of five), 36% were female and 64% were male. Further, 40% percent of the fathers had a high-school education or less, 50% had completed some form of college or earned a two-year degree, and 10% had earned a Bachelor’s degree. Additionally, 40% of the fathers were born in Mexico and had been in the U.S. for an average of 24 years (M = 24.14, SD = 14.45). Eight of the ten fathers were married or living with someone and two fathers reported being single. Finally, the majority of fathers (70%) reported speaking both Spanish and English and 30% reported only speaking Spanish. Noticeably, these two monolingual Spanish-speaking fathers had a middle-school education or less.

***Middle-to-high income mothers.*** Middle-to-high income mothers (*n* = 10) ranged in age from 23 to 35 years (M = 30.60, SD = 4.74). On average these mothers had one child (M = 1.20, SD = 0.42) under the age of five years old (M = 2.0 years, SD = 1.3). From the target children (i.e., under the age of five), 33% were female and 67% were male. Twenty percent of the mothers had completed some form of college, 20% had earned a Bachelor’s degree, and 60% had earned a Master’s degree or beyond. Additionally, only one of the mothers was born outside of the United States (i.e., Peru) and had been in the U.S. for 10 years. Nine of the ten mothers were married or living with someone and one mother reported being single. Finally, all mothers reported speaking both Spanish and English.

***Middle-to-high income fathers.*** Finally, middle-to-high income fathers (*n* = 10) ranged in age from 23 to 41 years (M = 33.50, SD = 5.72). On average, they had two children (M = 1.90, SD = 0.99) and at least one child was under the age of five years old (M = 2.3 years, SD = 1.4). From the target children (i.e., under the age of five), 38% were female and 62% were male. Sixty percent of the fathers had completed some form of college or earned a two-year degree and 40% had earned a Master’s degree or beyond. Additionally, only one of the fathers was born in Mexico and had been in the U.S. for 20 years. All fathers in this group reported being married or living with someone. Finally, 90% of the fathers reported speaking both Spanish and English, and one reported only speaking English.

### 2.2. Procedure

Eligible mothers and fathers were invited to participate in a semi-structured interview at a date and time that was most convenient for them. All interviews were conducted by the author of this article, a bilingual (Spanish–English) Latina researcher with prior experience working with Spanish-speaking Latine families. These interviews were conducted in a setting where the parent expressed feeling the most comfortable, with most choosing their home and a handful selecting a coffee shop or park and in the language of the parent’s choice, including Spanish, English, and a mix of Spanish and English. Interviews ranged in duration from 45 min to 1 h depending on the length of the response from the parent. At the start of the interview, the researcher provided the parent with a copy of the informed consent form and offered to discuss it with them and answer any questions or concerns. The form included a description of the study and the parent’s right to stop the interview at any time or opt out of answering a particular question. Parents were also asked for permission to audio-record and transcribe the interview. After the parents signed the informed consent form, the researcher turned on an audio-recording device and began the interview. When the interview was over, parents were compensated with a USD 10 Target gift card and a bilingual children’s book. All procedures and measures were approved by the Institutional Review Board at the University of California, Irvine, prior to data collection.

### 2.3. Measures

***Parents’ income category.*** Parents were asked to report on their: (1) total household annual income, (2) total number of people living in their household at least four days of the week, and (3) the number of these individuals who were minors and adults. Using this information, parental income level was determined by calculating their poverty index, which compared a family’s annual household income to an income threshold level that varied by family size and composition (i.e., number of children and adults).

The threshold levels are updated every year for inflation with the Consumer Price Index (U.S. Census Bureau, 2016). A family is considered to be living in poverty if their household annual income is less than the threshold level (U.S. Census Bureau, 2016). In their study, Brooks-Gunn et al. (1999) identified five income-to-needs ratios: (1) deep poverty (income-to-needs ratio less than 0.50), (2) poverty (income-to-needs ratio greater than or equal to 0.50, but less than 1.0), (3) near poverty (income-to-needs ratio between 1.0 and 1.5), low income (income-to-needs ratio between 1.5 and 2.0), and middle income (income-to-needs ratio greater than or equal to 2.0). However, I only identified two categories for this study: low income (income-to-needs ratio less than 2.0) and middle-to-high income (income-to-needs ratio equal to or greater than 2.0).

***Background questionnaire.*** Parents were asked to answer a 15-item background questionnaire created for this study. Questions asked parents to state their relationship with the target child(ren), as well as to identify their own and their child’s gender, age, and race/ethnicity, their income, the number of people living in their household, their education level, their marital status, their nationality, years living in the United States, and the language(s) the parent spoke.

***Semi-structured interview.*** The interview protocol was developed for this study and contained four sections. This manuscript focuses only on Section 1: parents’ beliefs about how mobile screen devices and social media supported and/or hindered their parenting. Parents were asked open-ended questions, such as “As a parent, how has having a smartphone or tablet helped you?” and “In what ways, if any, do you think these devices have made parenting more difficult?”. It is important to note that in answering the questions, I asked parents to think about their young children (i.e., under the age of five years old). To try and ensure that the questions were clear and interpreted as intended in both languages, extensive Spanish and English cognitive interviews were carried out with parents with similar background characteristics prior to data collection.

### 2.4. Qualitative Coding and Analysis

All audio-recordings were transcribed verbatim in their original language. Transcripts were coded in MAXQDA, using the original language of the interview. Pseudonyms were assigned to all participants to maintain confidentiality. A qualitative content analysis approach was employed using a combination of inductive and deductive strategies. Initial open coding allowed themes to emerge organically from the data, while deductive codes were informed by previous research on parenting and technology use. The author created an initial coding scheme using conceptual grouping of participants’ responses. Rather than organizing by demographic characteristics at the outset, themes were developed across interviews and later examined for patterns by income, gender, and language.

A concept-driven strategy guided the formation of the main categories, drawing on the research questions and recurring concepts in the data. Subcategories were developed through a data-driven process that grouped specific examples of how parents believed mobile screens supported or hindered parenting. The coding frame was refined through peer review: several independent undergraduate student researchers reviewed and proposed categories based on sample interview excerpts. Through comparison and discussion, a final coding scheme was established. Following this, all transcripts were recoded using the final codebook. Five trained doctoral students then reviewed a subset of excerpts and assigned them to categories, with flexibility to propose alternatives if needed. Frequencies of codes were extracted in MAXQDA to examine patterns across income levels, gender, and language background. A difference in theme prevalence by income or gender was noted if a theme appeared in at least twice as many participants in one group compared to another (i.e., a 2:1 ratio). Because only six participants were monolingual Spanish speakers and one was monolingual English-speaking, differences by language group were only discussed when none of the six monolingual Spanish speakers endorsed a theme and 30% or more of the 33 bilinguals did—or vice versa. Due to the small number of monolingual English speakers (*n* = 1), no comparisons were made between the father and other groups. Further, to enhance trustworthiness, the first author held regular peer debriefing meetings with research collaborators and used in-interview member checks by summarizing participants’ responses and asking for confirmation or clarification during the interview to ensure accurate interpretation. This trustworthiness strategy aligns with recommended practices in qualitative content analysis (Schreier, 2014) and iterative, concept-driven coding (Saldaña, 2003).

## 3. Results

A total of seven themes were identified that captured how socioeconomically and linguistically diverse Latine mothers and fathers of young children perceived mobile screen technologies as both supporting and complicating their parenting experiences. These themes were organized under three overarching functions that conceptualize mobile screen technologies as culturally situated tools operating within children’s microsystems:

Function 1: *Mobile Screen Devices as Parenting Tools for Access to Resources*
  - *Provide Access to Information* (theme 1)
  - *Provide Access to Social Support* (theme 2)
Function 2: *Mobile Screen Devices as Parenting Tools that Shape Parent–Child Interactions*
  - *Facilitate Teaching* (theme 3)
  - *Facilitate Parent–Child Bonding* (theme 4)
  - *Disrupt Parent–Child Interactions* (theme 5)
Function 3: *Mobile Screen Devices as Parenting Tools that Influence Parent and Child Emotional Regulation and Wellbeing*
  - *Aid and Complicate Managing Children’s Behavior* (theme 6)
  - *Provide Parental Emotional Relief and Stress* (theme 7)

Within this framework, parents play an active and primary role in determining how mobile screen technologies mediate their children’s everyday interactions with key adults (e.g., parents themselves), as well as with activities, and other objects and resources available to the child across key microsystems during early childhood (e.g., home, playground). Parents’ reported views and uses of mobile screen devices reflected their parenting goals, cultural values, and contextual constraints, and in turn shaped the quality and nature of children’s proximal processes—those everyday, reciprocal interactions that are foundational to early development. Importantly, all seven themes were represented across parent gender, income, and language groups. However, meaningful variation in subthemes emerged based on socioeconomic status, gender, and linguistic background. The themes are presented below by their corresponding function.

### 3.1. Function 1: Mobile Screen Devices as Parenting Tools for Access to Resources

***Access to Information.*** The vast majority of parents (95%) across income levels (*n* = 18 low, *n* = 20 middle-to-high), parent gender (*n* = 19 mothers, *n* = 19 fathers), and language groups (*n* = 5 Spanish-speaking; *n* = 33 English-speaking: 32 bilingual, 1 English only) reported using their mobile screen devices to instantly access parenting-related content. This included information about child health, developmental guidance, and learning strategies. As Carmen, a middle–high income mother with a PhD, explained: “*Um, so any kind of information from child health to like education to any question I have, it’s always my Smartphone where I go to*”.

However, meaningful differences emerged among Spanish-speaking parents with very low levels of formal schooling (*n* = 3). Two of the three parents with only an elementary school education did not use their devices to look up parenting or child-related information, though they did use them to help their older children with homework assignments. One of these parents, Esperanza (a low-income mother), described feeling uncertain about trusting online content: *“Me da miedo darle a mis niños cosas. Como nunca lo he usado para eso, en veces veo que dicen mira que esto es para la caída del pelo. Y en veces me digo, lo voy a hacer, pero después digo, ay no, ¿qué tal si llego a hacer algo a mis niños o algo?”* [*English translation:* “I’m afraid to give my kids things. Since I’ve never used it for that, sometimes I notice they say, look this is for hair loss. And sometimes I think I’ll try it but then I say, no—what if I end up doing something to my kids?”].

In contrast, the three Spanish-speaking parents with a middle-school education or higher reported using their screen devices to access information specifically related to their young children. This might suggest that formal schooling, rather than income or language alone, may play a greater role in shaping whether and how parents use mobile screen devices for informational purposes—though these patterns should be interpreted cautiously given the small number of monolingual participants.

Differences also emerged in the types and sources of information parents described seeking. Middle-to-high income parents and low-income mothers reported looking up a broader array of topics, while low-income fathers primarily searched for health-related information. Health information was also the most commonly searched topic among middle-to-high income parents (90%), though many also sought parenting advice (50%) and child development resources (25%). Similarly, half of low-income mothers reported searching for parenting guidance and child development tips, though none mentioned seeking health information. Further, parents with a high-school degree or higher often referenced using multiple sources (e.g., Google, forums), and several with graduate-level education mentioned reading research articles. Luis, a middle–high income father with a Master’s degree, explained the following: “*So I’m not really reading your .coms or stuff like that… my first go-to is if there’s something that I wanna read about… how is it being defined in the research*?”.

Two subthemes emerged within this broader theme of *Access to Information*. First, 17% of parents described mobile screens as particularly helpful during early parenthood when they had frequent questions. Daisy, a low-income mother with a high-school degree, shared that “*It’s been pretty helpful because I’m a young parent, I don’t know a lot… sometimes I don’t like asking for help from my mother-in-law, my mom. So I’d rather do my searching*”.

The second subtheme reflected the sense of empowerment some parents felt when they used the information they found online to advocate for their children. For example, Joshua, a middle–high income father with a 2-year college degree, said that “*I presented that [information] to the doctor and they said, yeah, you know what? You’re right. It would be a concern. So it’s kind of cool ‘cause it helped me diagnose what things she’s been going through already*”.

In summary, most parents—across income, gender, and language—used mobile devices to access parenting-related information. However, low-income fathers and parents with very low levels of formal schooling reported more limited use. At the same time, several new or younger parents described their devices as a critical source of guidance, and some parents across groups expressed feeling empowered as parents by the information they found.

***Access to Social Support*.** In total, 42% of parents discussed using their mobile screen devices to access social support from family or friends—most often through social media. This support was informational (e.g., advice, remedies), emotional (e.g., validation), or a combination of both (e.g., reassurance and concrete strategies). While the use of mobile screen devices for social support did not vary by income, differences seemed to emerge by gender and language. More mothers (*n* = 12) than fathers (*n* = 5) and more English-speaking parents (*n* = 17 bilingual, *n* = 1 monolingual) than monolingual Spanish-speaking parents (*n* = 1) reported seeking support via social media. Across all groups, however, direct phone calls with family remained a key mode of social connection and support. Most online social support was received through Facebook messages and, less commonly, comments on posts. Notably, the most frequently cited source of support—both online and offline—was the family (70%).

Further, several of the parents who did not use social media for support emphasized their preference for family-based or in-person support, citing proximity and trust as most valuable to them. Jorge, a middle–high income father who completed some form of college, explained that “*I’m just more comfortable… even if there’s a problem with her, I can just take her to my mom and show her what the symptoms are… she’ll compare it to one of us that it happened to*”. Some fathers also expressed discomfort discussing parenting concerns on social media. Even among parents who had previously sought support online, most stated they still preferred in-person support from family.

A smaller group of parents (*n* = 7) reported joining parenting groups or pages on Facebook—often in response to unique circumstances such as having a child with a medical condition or seeking solidarity about their parenting decisions. Gerardo, a low-income father with a 2-year degree, described joining a Facebook group for parents of children with kidney issues: “*I liked it… seeing what they were going through and what we’re going through… kind of what can work out better for ourselves and for our children*”. Similarly, Yesenia, a low-income mother with a Bachelor’s degree, shared how online validation helped her feel confident in her decision to breastfeed: “*Some people around me put me down… but sometimes I would read stuff that people would post… and it made me feel more reinforced in what I was doing*”. Notably, none of the parents who sought online parenting groups were monolingual Spanish speakers, and no Spanish-speaking parents discussed facing unique parenting challenges requiring outside support. In sum, while family remained the primary source of social support across all groups, a subset of parents—especially mothers and English-speaking parents—used their devices to expand their support networks during periods of stress or feelings of isolation.

### 3.2. Function 2: Mobile Screen Devices as Parenting Tools That Shape Parent–Child Interactions

This function captures parents’ experiences related to their mobile screen technologies both strengthening and hindering their daily interactions with their children. Below are more details about the three themes informing this function.

***Facilitate Parent–Child Bonding.*** Nearly all parents (97%) reported co-using mobile screen devices with their children, and 23% elaborated on how these moments were meaningful bonding experiences for them. This enjoyment was evenly distributed across income (*n* = 5 low-income, *n* = 4 middle-to-high income) and gender (*n* = 4 mothers, *n* = 5 fathers). Parents of children as young as 3 months of age and up to 4 years old described using mobile screens to share laughs, build on their child’s interests, and spend quality time together. For instance, Eric, a middle–high income father with some form of college education, explained that “*I feel like I connect with her in what she likes and I could just see more of what she enjoys, like she loves My Little Pony*”. Similarly, Daisy, a low-income mother with a high-school degree, recalled a bonding moment with her child: “*all of the sudden he just starts kissing me, hugging me… he starts acting like a baby*”.

Although all Spanish-speaking parents (*n* = 6) also reported co-using devices with their children, none spontaneously discussed enjoying it—though two had very young infants (1 month old) and reported using the device primarily to Facetime relatives in Mexico. These findings might suggest that while most parents used mobile screen devices to engage with their children, this type of use varied based on child age.

***Facilitate Teaching.*** Thirty-five percent of parents across income (*n* = 7 low, *n* = 7 middle-to-high), gender (*n* = 7 mothers, *n* = 7 fathers), and linguistic groups (*n* = 2 Spanish-speaking; *n* = 13 English-speaking: 12 bilingual, 1 monolingual English-speaking) described using mobile screen devices to support their child’s learning. These devices were often used to illustrate academic concepts further by capitalizing on children’s interests (e.g., dinosaurs) and model desired behaviors (e.g., potty training). Anthony, a low-income father who completed some form of college, shared that “*my son right now is into like dinosaurs so I find videos on YouTube and I show it to him… he’s like that’s a T-Rex, that’s a Stegosaurus*”.

A notable subtheme among bilingual parents was the intentional use of mobile screen devices to support their children’s Spanish-language development. Parents reported searching for Spanish-language songs and videos on YouTube. For example, Ricardo, a middle–high income father with a PhD, explained that “*I put songs in Spanish for the kids… they start picking up on Spanish words and phrases… they know that ‘vamos a’ means we’re going somewhere*”. While Spanish-speaking parents did not mention actively seeking Spanish-language media, many noted that their children were learning English through content viewed on the screen device.

***Disrupt Parent–Child Interactions.*** While all parents acknowledged the benefits of mobile screen devices, nearly half (47%) also noted that these technologies sometimes disrupted interactions with their children. This concern was expressed more often among middle-to-high income parents (*n* = 13) compared to low-income parents (*n* = 7). Jennifer, a middle–high income mother with some form of college education, described that “*we’re not really engaging with our daughter the right way when we have our phones… when we’re playing play pretend, like it’s not the same*”. Notably, none of the four Spanish-speaking parents with less than a high-school education expressed concern about disrupted interactions. In contrast, within the low-income group, the two mothers and one father with a Bachelor’s degree did report concerns about disruption. This suggests that perceptions of device interference may be linked more closely to formal schooling than income or language. For example, Martin, a low-income, Spanish-speaking father with a middle-school education, stated that “*Si tengo tiempo, lo uso y si no. Primero está el trabajo, mi familia… El teléfono no es de que me voy a morir*” [Translation: “If I have time, I’ll use it, if I don’t, I won’t. My priority is work, my family… I’m not going to die if I don’t use my phone”].

Together, these findings highlight the nuanced ways in which mobile screen devices shaped parent–child interactions. For many parents, co-use of screens was a means of connecting with their children and fostering their learning. At the same time, concerns about distraction and disrupted engagement—especially among more formally highly educated parents—illustrate the complex role these technologies play in parents’ childrearing experiences.

### 3.3. Function 3: Mobile Screen Devices as Parenting Tools That Influence Parent and Child Emotional Regulation and Wellbeing

***Aid and Complicate Managing Children’s Behavior.*** One of the most consistent benefits parents (90%) associated with mobile screen technologies was their effectiveness in helping them manage their young children’s behavior during challenging situations. This theme was prevalent among parents across all child age groups (3 months through 4 years), income levels (*n* = 16 low-income, *n* = 20 middle-to-high income), genders (*n* = 18 mothers, *n* = 18 fathers), and language groups (*n* = 2 Spanish-speaking; *n* = 32 English-speaking: *n* = 31 bilingual, *n* = 1 monolingual English-speaking). Across groups, parents described sometimes relying on mobile screen devices to soothe, distract, or entertain their child when they were fussy, particularly in public settings. These devices were also helpful in distracting their children when parents needed to complete chores, or when they simply needed a break. For example, Lorena, a low-income mother with a high-school degree, explained that “*For the baby, when he’s behaving bad… if we’re out though! Not at home. Like if we are out and about… Interviewer: Why more outside? Lorena: Than inside the house? Cuz at home I could control him and outside he screams or people look at you and the first reaction is, oh here’s my phone so you could be quiet*”.

Somewhat similarly, an additional subtheme—unique to middle-to-high income parents and low-income parents who were enrolled in college—involved using the mobile screen devices to entertain their child during remote work or study time when no alternative care was available. Despite the widespread use of mobile screens for behavioral support, most parents stressed that they also used non-digital means of entertainment first (e.g., toys) and tended to turn to mobile screen devices when those options were not feasible or successful.

Additionally, nearly half of all parents (47%) also reported using mobile screen devices to reward or discipline their children. These parents—distributed across income (*n* = 9 low-income, *n* = 10 middle-to-high income), gender (*n* = 8 mothers, *n* = 11 fathers), and language groups (*n* = 2 Spanish-speaking; *n* = 17 English-speaking: *n* = 16 bilingual, *n* = 1 monolingual)—emphasized that this strategy was only applied to toddlers or preschoolers. As Aniceto, a low-income father with an elementary school education, described, “*A veces cuando no se estan portando bien, les remuevo el celular. Y ya una vez que los miro que estan calmados entonces lo volvemos a intentar de nuevo*” (“Sometimes I remove the smartphone when they’re not behaving well. Once I see that they calmed down, then we try it again”). At the same time, 45% of parents also expressed concern about the unintended behavioral challenges that arose due to their children’s growing dependence on the screen devices. These parents were evenly distributed across income (*n* = 10 low-income, *n* = 8 middle-to-high income) and gender (*n* = 10 mothers, *n* = 8 fathers). Specifically, they described experiences such as children pestering them for the device or having tantrums when it was taken away. Some parents also shared that sometimes fights between siblings over using these mobile screen devices would arise. Carlos, a middle–high income father with a Master’s degree, explained that “*The thing is, we don’t let him use it [tablet] as much because whenever we do take it away from him, he throws the biggest fit. He starts crying and kicking, so we always have to remind him, like hey, once we get to the car, like we’re gonna take the phone away. Um… that doesn’t always work*”. These findings suggest that while mobile screen devices served as practical tools for helping parents manage their children’s behavior, they also introduced new stressors around emotional regulation, particularly for young children.

***Provide Parental Emotional Relief and Stress.*** Parents also shared how mobile screen technologies affected their own emotional wellbeing—both positively and negatively. Overall, 75% of parents described feeling positive emotions when they used their mobile screen devices, and 50% also shared experiencing negative emotions. These emotional impacts appeared to differ based on parents’ socioeconomic, educational, and linguistic backgrounds. Across income (*n* = 15 low-income, *n* = 16 middle–high income), gender (*n* = 16 mothers, *n* = 15 fathers), and linguistic groups (*n* = 4/6 Spanish-speaking; *n* = 27 English-speaking: *n* = 26 bilingual, *n* = 1 monolingual), many parents reported that mobile screen devices helped them relax, manage boredom, and feel validated as parents. For example, Eric, a middle–high income father with some form of college education, said that “*It lets us know we’re not alone in how we feel, as much as we don’t wanna be frustrated or stressed out, it still happens, you know? But I mean it happens to everyone, we’re not alone. We’re not the only parents that feel that way. We just wanna make sure our child is ok, you know? So that’s a great relief, at least for me it’s like ok we’re not bad parents*”. Slight group differences emerged within these positive emotions. A small number of middle-to-high income parents (*n* = 4 mothers, *n* = 3 fathers) and low-income mothers (*n* = 3) noted feeling relief or validation after finding information or receiving emotional support online. Only one low-income father described this kind of emotional benefit. These parents all had a high-school education or higher and were not monolingual Spanish speakers.

Further, more than half (55%) of all parents described using mobile screen devices to manage stress or escape boredom. These parents were distributed across income (*n* = 13 low, *n* = 9 middle–high) and gender (*n* = 12 mothers, *n* = 10 fathers), and notably included Spanish-speaking parents with low levels of formal schooling. Alvaro, a low-income father, explained that “*Cuando a veces me siento como aburrido o triste o desesperado, simplemente con ver algo divertido borra un poco el sentimiento, si mas que nada es el sentimiento*” (“Sometimes when I feel bored or sad or anxious, I erase some of those feelings by simply looking at something funny, yes more than anything it’s the feeling”). Still, 50% of parents also described negative emotions tied to their use of mobile screens. These parents disproportionately had a middle-to-high income (*n* = 15), with fewer low-income parents (*n* = 5) reporting similar experiences. These negative emotions often stemmed from feeling stressed or guilty about their screen use—either for themselves or for their child. For instance, Marcos, a middle–high income father with a two-year degree, noted that “*My worries are on the phone. You know, like tasks that you have to get done and stuff like that… events are coming up or your loads schedules… But I guess it depends on the industry that you’re in*”.

These feelings of guilt were particularly common among middle-to-high income mothers (*n* = 8), followed by middle-to-high income fathers (*n* = 4) and low-income parents (*n* = 2 mothers, *n* = 3 fathers). Yaritza, a middle–high income mother with a Master’s degree, expressed that “*I don’t wanna be that work mom for her. You know? Where it’s like, she’s talking and I’m going yeah, yeah, yeah, oh good, oh good, good. You know? Cuz I feel like she picks up on it and so I feel so bad*!” In contrast, parents who did not report feeling guilty often cited having clear boundaries around device use or not caring about external judgment. Lupe, a low-income mother with some form of college education, stated that “*Como tenemos limites, entonces pienso que estamos bien. Y al fin y al cabo es mi manera pensar y de hacer las cosas* [laughs]” (“Since we have limits, I think we are okay. And after all, it’s my way of thinking and doing things”).

In sum, while mobile devices often provided emotional relief and affirmation for parents, they also introduced feelings of guilt and stress—particularly among parents with more access to digital work or higher educational expectations for themselves. These complex emotional effects highlight the dual role of mobile screens in shaping the parenting environment and influencing family wellbeing.

## 4. Discussion

This study examined how socioeconomically and linguistically diverse Latine mothers and fathers of young children perceived the role of mobile screen technologies in their everyday parenting experiences. Grounded in the revised bioecological systems model (Vélez-Agosto et al., 2017), these findings extend prior research by conceptualizing mobile devices as culturally situated tools that mediate parent–child interactions and shape children’s environments across home and community microsystems. Rather than passive recipients of digital technologies, parents in this study described intentional, adaptive uses of mobile screen devices that reflected their parenting goals, cultural values, and available resources. At the same time, parents also acknowledged how the same tools that provided them with parenting support could unintentionally disrupt family interactions and their children’s wellbeing—illustrating the complex, multifaceted role of mobile screen technologies in parenting.

The finding that nearly all parents used mobile devices to access information about child health, development, or parenting strategies supports prior research on the utility of mobile screen devices as informational tools (Kabali et al., 2015). However, this study expands that work by illustrating how patterns of information-seeking differed by parental level of formal schooling and gender. For example, low-income fathers reported narrower searches focused on child health, while more educated parents—regardless of income—described feeling empowered by the breadth and credibility of information they accessed. These findings align with the idea that proximal processes are shaped not just by individual agency but by the interaction between individuals and their sociocultural context (Vélez-Agosto et al., 2017).

Similarly, the use of mobile screen devices to access social support—particularly among mothers and English-speaking parents—builds on past work highlighting the social affordances of screen media (Radey & Randolph, 2009; Wartella et al., 2013). Yet few studies have documented how diverse Latine parents navigate informal and online networks for reassurance and parenting advice. This reliance on family—whether in-person or digitally mediated—reflects the cultural value of *familismo*, which emphasizes strong emotional bonds, loyalty, and mutual support among family members (Campos et al., 2008; Calzada, 2010). For many Latine families, parenting decisions are situated within a broader network of relatives who provide emotional and practical guidance. While some participants described turning to online spaces, the consistent role of family as the main source of parenting support highlights how culturally grounded caregiving practices persist even in increasingly digital contexts. The present findings show that some parents—especially those experiencing unique parenting challenges—turned to online parenting communities, reflecting an intentional search for culturally relevant and context-specific support. This emphasizes the importance of viewing mobile screen technology use through a strength-based lens that centers parents’ agentic and adaptive strategies in response to structural constraints.

Findings related to the shaping of parent–child interactions also reveal novel contributions. Consistent with work by Blum-Ross and Livingstone (2016), many parents described co-using mobile screens with their children as a bonding or educational experience, particularly to support their child’s bilingual development—an underexamined goal in the literature. Notably, parents actively curated content to reinforce cultural values and foster dual-language learning, illustrating how mobile screen technologies can be integrated into cultural transmission and parent-led teaching. This supports Vélez-Agosto et al.’s (2017) call to view digital practices as embedded in culturally specific routines and goals, rather than universal or deficit-based.

Importantly, parents across income and linguistic groups also recognized the potential for mobile screens to interfere with responsive interactions. These findings align with previous concerns about “technoference” among the majority of middle-class White mothers (McDaniel & Radesky, 2018), but unlike most studies that frame such interference as a parental shortcoming, the present study underscores parents’ self-awareness and contextual reasoning. For instance, high-resource mothers expressed guilt due to work-related phone use, while lower-resource parents emphasized their efforts to prioritize family time despite limited availability. This highlights the need to consider how cultural expectations, parental role demands, and social surveillance shape perceptions of appropriate technology use.

Themes related to emotional regulation and behavior management further illustrate the duality of mobile screens. While nearly all parents used devices to soothe or occupy their children—especially in public or high-stress situations—many also described negative consequences, including child dependence, tantrums, and sibling conflict. These accounts mirror prior findings (Radesky et al., 2016) but also demonstrate parents’ nuanced reflections on both the benefits and tradeoffs of these devices. Rather than dismissing these practices as problematic, this study reveals the deliberative ways parents weigh their decisions within structurally constrained environments, reinforcing the value of ecological and strength-based frameworks.

Finally, the emotional complexity associated with screen use—particularly feelings of guilt among middle- to high-income mothers—extends the literature on parental wellbeing and screen time. While some research has linked digital media use to parent stress (Coyne et al., 2017), the current study adds important context by showing how feelings of guilt were mediated by social class, perceived social judgment, and the parent’s sense of control. These insights underscore the importance of considering how societal discourses about “good parenting” intersect with cultural and structural realities in shaping parents’ emotional responses to technology use.

## 5. Limitations and Implications

Several limitations should be considered when interpreting the findings of this study. First, although the sample was diverse in terms of socioeconomic status, gender, and language, it was not nationally representative and included a small number of monolingual Spanish-speaking parents. As a result, the language-related patterns observed—particularly among Spanish-speaking parents with limited formal schooling—should be interpreted with caution. Future studies with larger samples of monolingual parents across various dialects and regions are needed to examine linguistic variation in greater depth.

Second, the majority of parents in this sample were married or living with a partner, which might have implications for the type and level of support families receive and their subsequent use of screen media. Hence, the results from this study might not generalize to single-parent households, and future studies should investigate whether and how different household compositions relate to the level of support families receive and how this might relate to families’ patterns of screen media usage and beliefs.

Third, this study relied on self-reported data collected through semi-structured interviews, which may be subject to recall bias or social desirability due to the sensitive topic and parents’ sense of being judged. However, the depth and nuance of parents’ responses suggest that participants felt comfortable reflecting on both the positive and negative aspects of their mobile device use in their parenting roles. Moreover, the qualitative design allowed for rich insights into culturally embedded caregiving practices that are often overlooked in survey-based research.

Fourth, this study did not systematically collect data on children’s participation in early childhood education or care settings, which may have influenced parenting practices around screen use. Future research should consider how caregiving contexts—including home-based care, center-based care, or mixed arrangements—shape how parents use mobile technologies in their day-to-day interactions with young children

Fifth, although both mothers and fathers were included, this study did not explicitly examine the dynamics between co-parents or shared decision-making around screen use. Future research could explore how screen-related parenting practices are negotiated within households, including intergenerational influences and extended family dynamics—especially given the importance of familial networks in Latine communities.

Despite these limitations, this study offers several important implications. Conceptually, it advances the field by integrating a culturally grounded bioecological framework to understand how mobile screen technologies operate within the caregiving environments of socioeconomically and linguistically diverse Latine families with young children. Rather than framing screen use as inherently problematic, this study highlights parents’ intentional and adaptive uses of mobile screen technologies in ways that reflect their cultural values, caregiving goals, and structural realities.

Practically, the findings point to the importance of developing culturally responsive guidance for families navigating screen use in early childhood. Interventions and educational materials should move beyond one-size-fits-all approaches and instead recognize the nuanced ways parents use technology to bond with, teach, and regulate their children. For example, practitioners might support families in identifying high-quality, culturally relevant digital content, or in developing shared media routines that promote interaction and emotional connection.

Finally, the findings underscore the need for policy and public discourse to shift away from deficit-based narratives about screen use in low-income or ethnoracially minoritized households. Rather than only assuming harm, policymakers and educators should consider how mobile screen technologies can serve as empowering tools for parents—especially when used by parents with clear intentions and in response to contextual barriers. Supporting Latine families means acknowledging the resourcefulness they bring to their parenting practices and co-constructing tools that align with their lived realities.

## Figures and Tables

**Table 1:** Parent demographic characteristics summarized by income and gender.

Group	*n*	Age Range	*M* Age	Self-Reported Language(s) Spoken	Country of Birth for Foreign-Born Parents	Formal Schooling Level
Low-Income Mothers	10	22–36	28.77	Only Spanish: (*n* = 3) Bilingual (English and Spanish): (*n* = 7)	Mexico: (*n* = 6) Ecuador: (*n* = 1)	High School or less: (*n* = 6) Some College: (*n* = 2) 4-Year Degree: (*n* = 2)
Low-Income Fathers	10	26–45	31.5	Only Spanish = 3 Bilingual (English and Spanish) = 7	Mexico: (*n* = 4)	High School or less: (*n* = 4) Some College: (*n* = 5) 4-Year Degree: (*n* = 1)
Mid/High-Income Mothers	10	23–35	30.6	Bilingual (English and Spanish) = 10	Mexico: (*n* = 1)	Some College: (*n* = 2 4-Year Degree: (*n* = 2) Master’s Degree or Higher (*n* = 6)
Mid/High-Income Fathers	10	23–41	33.5	Bilingual (English and Spanish) = 9 Only English = 1	Mexico: (*n* = 1)	Some College: (*n* = 6) Master’s Degree or Higher (*n* = 4)

Note. All participants self-identified as Latine and were parents of at least one child between 0 and 5 years old. Language categories are mutually exclusive.

## Data Availability

Data supporting the findings of this study are available from the corresponding author upon reasonable request.

## References

[B1-behavsci-15-01139] Blum-Ross A., Livingstone S. (2016). Families and screen time: Current advice and emerging research.

[B2-behavsci-15-01139] Bronfenbrenner U. (2005). Making human beings human: Bioecological perspectives on human development.

[B3-behavsci-15-01139] Brooks-Gunn J., Duncan G. J., Aber J. L., Brooks-Gunn D. J., Duncan G. J., Aber J. L. (1999). Cognitive and emotional outcomes for children in poverty. Neighborhood poverty: Context and consequences for children.

[B4-behavsci-15-01139] Cabrera N. J., Bradley R. H. (2012). Latino fathers and their children. Child Development Perspectives.

[B5-behavsci-15-01139] Calzada E. J. (2010). Bringing culture into parent training with Latinos. Cognitive and Behavioral Practice.

[B6-behavsci-15-01139] Campbell S. W., Ling R., Bayer J., Oliver M. B., Raney A. (2014). The structural transformation of mobile communication: Implications for self and society. Media and social life.

[B7-behavsci-15-01139] Campos B., Dunkel Schetter C., Abdou C. M., Hobel C. J., Glynn L. M., Sandman C. A. (2008). Familialism, social support, and stress: Positive implications for pregnant Latinas. Cultural Diversity and Ethnic Minority Psychology.

[B8-behavsci-15-01139] Center on the Developing Child at Harvard University (2016). From best practices to breakthrough impacts: A science-based approach to building a more promising future for young children and families.

[B9-behavsci-15-01139] Common Sense Media (2017). The common sense census: Media use by kids age zero to eight.

[B10-behavsci-15-01139] Council on Communications and Media (2016). Media and young minds. Pediatrics.

[B11-behavsci-15-01139] Coyne S. M., Radesky J., Collier K. M., Gentile D. A., Linder J. R., Nathanson A. I. (2017). Parenting and digital media. Pediatrics.

[B12-behavsci-15-01139] Criss S., Woo Baidal J. A., Goldman R. E., Perkins M., Cunningham C., Taveras E. M. (2015). The role of health information sources in decision-making among Hispanic mothers during their children’s first 1,000 days of life. Maternal and Child Health Journal.

[B13-behavsci-15-01139] Duggan M., Lenhart A. (2015). Parents and social media: Mothers are especially likely to give and receive support on social media.

[B14-behavsci-15-01139] Fuller B., Lizárraga J. R., Gray J. H. (2015). Digital media and latino families: New channels for learning, parenting, and local organizing. Joan ganz cooney center at sesame workshop.

[B15-behavsci-15-01139] Glick F., Saiyed A., Kutlesa N. (2022). Now you see me, now you don’t: Children learn grammatical choices during online socially contingent video and audio interactions. Proceedings of the 46th Boston university conference on language development.

[B16-behavsci-15-01139] Hiniker A., Sobel K., Suh H., Sung Y., Lee C. P., Kientz J. A. (2015). Texting while parenting: How adults use mobile phones while caring for children at the playground. 33rd Annual SIGCHI Conference on Human Factors in Computing Systems (CHI ’15).

[B17-behavsci-15-01139] Kabali H. K., Irigoyen M. M., Nunez-Davis R., Budacki J. G., Mohanty S. H., Leister K. P., Bonner R. L. (2015). Exposure and use of mobile media devices by young children. Pediatrics.

[B18-behavsci-15-01139] Katz J. E. (2002). Perpetual contact: Mobile communication, private talk, public performance.

[B19-behavsci-15-01139] Kildare C. A., Middlemiss W. (2017). Impact of parents’ mobile device use on parent–child interaction: A literature review. Computers in Human Behavior.

[B20-behavsci-15-01139] Kushlev K., Dunn E. W. (2019). Smartphones distract parents from cultivating feelings of connection when spending time with their children. Journal of Social and Personal Relationships.

[B21-behavsci-15-01139] Lauricella A. R., Ellerbe E., Wartella E., Gee E., Takeuchi L., Wartella E. (2018). Digital media as a parenting support tool for Hispanic families in the United States. Children and families in the digital age.

[B22-behavsci-15-01139] Mallawaarachchi S. R., Hooley M., Sutherland Smith W., Horwood S. (2022). “You’re damned if you do, you’re damned if you don’t”: A qualitative exploration of parent motives for provision of mobile screen devices in early childhood. BMC Public Health.

[B23-behavsci-15-01139] Mann S., Calvin A., Lenhart A., Robb M. B. (2025). The common sense census: Media use by kids zero to eight, 2025. *Common Sense Media*.

[B24-behavsci-15-01139] McDaniel B. T., Radesky J. S. (2018). Technoference: Parent distraction with technology and associations with child behavior problems. Child Development.

[B25-behavsci-15-01139] Neuman S. B., Celano D. (2012). Giving our children a fighting chance: Poverty, literacy, and the development of information capital.

[B26-behavsci-15-01139] Ochoa W., McWayne C. M., Díaz Lara G., Williams-Johnson M., Rickert N. P. (2024). Leveraging mobile screen technologies to foster a home-to-school approach for family engagement among Latine families with young children. Critical analysis of parental involvement in school: Working with families across sociocultural contexts.

[B27-behavsci-15-01139] Ochoa W., Reich S. M. (2020). Parents’ beliefs about the benefits and detriments of mobile screen technologies for their young children’s learning: A focus on diverse Latine mothers and fathers. Frontiers in Psychology.

[B28-behavsci-15-01139] Ochoa W., Reich S. M., Farkas G. (2020). The observed quality of caregiver–child interactions with and without a mobile screen device. Academic Pediatrics.

[B29-behavsci-15-01139] Pew Research Center (2017a). Smartphones help blacks, Hispanics bridge some—But not all-digital gaps with Whites.

[B30-behavsci-15-01139] Pew Research Center (2017b). How the U.S. hispanic population is changing.

[B31-behavsci-15-01139] Pew Research Center (2024). Mobile fact sheet. *Pew Research Center*.

[B32-behavsci-15-01139] Radesky J. S., Christakis D. A. (2016). Increased screen time: Implications for early childhood development and behavior. Pediatric Clinics of North America.

[B33-behavsci-15-01139] Radesky J. S., Eisenberg S., Kistin C. J., Gross J., Block G., Zuckerman B., Silverstein M. (2016). Overstimulated consumers or next-generation learners? Parent tensions about child mobile technology use. The Annals of Family Medicine.

[B34-behavsci-15-01139] Radesky J. S., Kistin C. J., Zuckerman B., Nitzberg K., Gross J., Kaplan-Sanoff M., Augustyn M., Silverstein M. (2014). Patterns of mobile device use by caregivers and children during meals in fast food restaurants. Pediatrics.

[B35-behavsci-15-01139] Radey M., Randolph K. A. (2009). Parenting sources: How do parents differ in their efforts to learn about parenting. Family Relations.

[B36-behavsci-15-01139] Reich S. M., Liu Y., Tulagan N., Martin E., Dahlin M., Cabrera N. (2023). Applying a family stress model to understand U.S. families’ patterns of stress, media use, and child behavior during the COVID-19 pandemic. Journal of Children and Media.

[B37-behavsci-15-01139] Rideout V. J. (2014). Learning at home: Families’ educational media use in America. A report of the families and media project.

[B38-behavsci-15-01139] Rideout V., Robb M. B. (2020). The common sense census: Media use by kids age zero to eight, 2020. *Common Sense Media*.

[B39-behavsci-15-01139] Saldaña J. (2003). The coding manual for qualitative researchers.

[B40-behavsci-15-01139] Schreier M., Flick U. (2014). Qualitative content analysis. The SAGE handbook of qualitative data analysis.

[B41-behavsci-15-01139] Sergi K., Gatewood R., Elder A., Xu J. (2017). Parental perspectives on children’s use of portable digital devices. Behaviour & Information Technology.

[B42-behavsci-15-01139] The Aspen Institute (2023). https://www.aspeninstitute.org/blog-posts/a-glance-at-the-latino-digital-gap-and-the-need-for-device-equity/.

[B43-behavsci-15-01139] Thompson D. A., Jimenez-Zambrano A. M., Ringwood H., Tschann J. M., Clark L. (2023). Parenting a toddler in the era of pervasive screens: Interviews with low-income Mexican American parents. International Journal of Environmental Research and Public Health.

[B44-behavsci-15-01139] U.S. Census Bureau (2016). Income and poverty in the United States: 2016 *(Current Population Reports, P60-259)*.

[B45-behavsci-15-01139] U.S. Census Bureau (2023). Hispanic heritage month: 2023.

[B46-behavsci-15-01139] Valencia R. R. (2010). Dismantling contemporary deficit thinking: Educational thought and practice.

[B47-behavsci-15-01139] Vélez-Agosto N. M., Soto-Crespo J. G., Vizcarrondo-Oppenheimer M., Vega-Molina S., García Coll C. (2017). Bronfenbrenner’s bioecological theory revision: Moving culture from the macro into the micro. Perspectives on Psychological Science.

[B48-behavsci-15-01139] Wartella E., Rideout V., Lauricella A. R., Connell S. L. (2013). Parenting in the age of digital technology: A national survey.

